# Magnetic resonance imaging of the erector spinae muscles in Duchenne muscular dystrophy: implication for scoliotic deformities

**DOI:** 10.1186/1748-7161-3-21

**Published:** 2008-12-29

**Authors:** Gnahoua Zoabli, Pierre A Mathieu, Carl-Éric Aubin

**Affiliations:** 1 Research Centre, Sainte-Justine University Hospital Centre, University of Montreal, 3175, Chemin de la Côte-Sainte-Catherine, Montréal, Québec, H3T 1C5, Canada; 2 Biomedical Engineering Institute, University of Montréal, C.P. 6128 succursale Centre-ville, Montréal, QC H3C 3J7, Canada; 3 Department of Mechanical Engineering, École Polytechnique, University of Montreal, C.P. 6079, succursale Centre-ville, Montréal, QC, H3C 3A7, Canada

## Abstract

**Background:**

In Duchenne muscular dystrophy (DMD), the muscular degeneration often leads to the development of scoliosis. Our objective was to investigate how anatomical changes in back muscles can lead to scoliosis. Muscular volume and the level of fat infiltration in those muscles were thus evaluated, in non-scoliotic, pre-scoliotic and scoliotic patients. The overlying skin thickness over the apex level of scoliotic deformations was also measured to facilitate the interpretation of electromyographic signals when recorded on the skin surface.

**Methods:**

In 8 DMD patients and two healthy controls with no known muscular deficiencies, magnetic resonance imaging (MRI) was used to measure continuously at 3 mm intervals the distribution of the erector spinae (ES) muscle in the T8-L4 region as well as fat infiltration in the muscle and overlying skin thickness: four patients were non-scoliotic (NS), two were pre-scoliotic (PS, Cobb angle < 15°) and two were scoliotic (S, Cobb angle ≥ 15°). For each subject, 63 images 3 mm thick of the ES muscle were obtained in the T8-L4 region on both sides of the spine. The pixel dimension was 0.39 × 0.39 mm. With a commercial software, on each 12 bits image, the ES contour on the left and on the right sides of the spine were manually determined as well as those of its constituents i.e., the iliocostalis (IL), the longissimus (LO) and the spinalis (SP) muscles. Following this segmentation, the surfaces within the contours were determined, the muscles volume were obtained, the amount of fat infiltration inside each muscle was evaluated and the overlying skin thickness measured.

**Findings:**

The volume of the ES muscle of our S and PS patients was found smaller on the convex side relative to the concave one by 5.3 ± 0.7% and 2.8 ± 0.2% respectively. For the 4 NS patients, the volume difference of this muscle between right and left sides was 2.1 ± 1.5% and for the 2 controls, it was 1.4 ± 1.2%. Fat infiltration for the S and the PS patients was larger on the convex side than on the concave one (4.4 ± 1.6% and 4.5 ± 0.7% respectively) and the difference was more important near the apex. Infiltration was more important in the lateral IL muscle than in the medial SP and it was always larger near L2 than at any other spinal level. Fat infiltration was much more important in the ES for the DMD patients (49.9% ± 1.6%) than for the two controls (2.6 ± 0.8%). As for the overlying skin thickness measured near the deformity of the patients, it was larger on the concave than on the convex side: 14.8 ± 6.1 vs 13.5 ± 5.7 mm for the S and 10.3 ± 6.3 vs 9.8 ± 5.6 mm for the PS.

**Interpretation:**

In DMD patients, our results indicate that a larger replacement of muscles fibers by fat infiltration on one side of the spine is a factor that can lead to the development of scoliosis. Efforts to slow such an infiltration on the most affected side of the spine could thus be beneficial to those patients by delaying the apparition of the scoliotic deformation. In addition to anatomical considerations, results obtained from the same patients but in experiments dealing with electromyography recordings, point to differences in the muscular contraction mechanisms and/or of the neural input to back muscles. This is similar to the adolescent idiopathic scoliosis (AIS) where a role of the nervous system in the development of the deformation has also been suggested.

## Background

Duchenne muscular dystrophy (DMD) is a severe genetic disease affecting one in 3500 boys [1]. Due to lack of dystrophin, muscle fibers are susceptible to mechanical damage leading to their replacement with fat tissue [2]. As muscular weakness progresses, most patients will experience falls resulting in disabling extremity fractures [3] and some of them will also develop a scoliosis [4]. While many researches are related to the genetic aspects of the disease [5] and to muscle damage repair [6], investigations have also been carried on the identification of anatomical changes associated with the disease.

For example, it is possible with ultrasound (US) imaging to monitor DMD progression by observing the pathologic changes of skeletal muscle fibers, the level of fat infiltration and the effect of a therapy [7]. With this imaging modality, the volume of superficial muscles can be estimated both at rest and under contraction)[8]. To assess fat infiltration in DMD back muscles, computerized tomography (CT) can also be used [9]. For scoliotic DMD patients, Stern and Clark [10] reported that fat infiltration was more important on the concave side of their deformity. These authors found that the difference was proportional to the severity of the deformity and was correlated with the progression of the DMD within a 6-month period. This was not the case for the non-scoliotic patients where the amount of muscle fibers replaced by fat was found similar on both sides of the spine. Since CT imaging modality implies radiation to the patient, magnetic resonance imaging (MRI) is getting more often used [11]. When body composition of DMD patients was evaluated with MRI [12], the mean fat mass represented 31.6 ± 17.0% of the body weight which is significantly higher than the 15.5 ± 12.1% obtained from growth charts. While MRI is capable of producing reliable results, processing of the collected images was lengthy few years ago but this has changed appreciably. For instances, with automatic segmentation methods, subcutaneous fat could be separated from muscle tissue within 2 s with an error < 3% [13].

Using MRI, the objective in the present study was to measure, along the spine, the volume and the level of fat infiltration in back muscles of DMD patients in order to better understand how the dystrophy affects those muscles. The overlying skin thickness over those muscles was also studied since it affects the characteristics of the surface electromyographic (EMG) signals recorded over those muscles.

## Materials and methods

Eight male DMD patients of 10.9 to 15.3 years old participated in this study. As shown in Table 1, four of them were non-scoliotic (NS), two were pre-scoliotic (PS, Cobb angle < 15°, convexity at the right) and two were scoliotic (S, Cobb angle ≥ 15°, convexity at the left). Their age, height and weight are shown as well as the number of months where they received deflazacort (DFZ) medication. P2 to P7 were still on that medication while P1 and P8 had stopped it at the time of the MRI acquisition. Their clinical mobility was assessed (last column of Table 1) according to the classification of Gibson and Wilkins [14]: 1- independent for a short distance, 2- assistance to standing, difficulty walking, 3- independent with brace, 4- walks with brace – support needed for balance, 5- wheelchair dependent – can move chair long distances, 6- wheelchair dependent – can move chair short distance, 7- limited to use of electric chair, 8- totally dependent. All patients were using a wheelchair, even those with a clinical mobility less than 5. As controls, two healthy boys of 9 and 10 years (C1, C2) with similar body mass index but without any known musculo-squelettal problem were included in the study. The experimental protocol was approved by the ethic committee of the hospital and a written consent was obtained from each patient as well as from each control.

**Table 1:** Information on the controls (C1 and C2) and on the DMD patients (P1 to P8) classified according to their Cobb angle.

Subjects	Cobb angle (°)	Apex level, Convexity	Group	Age (yrs)	Height (m)	Weight (kg)	BMI (kg/m^2^)	DFZ duration (months)	Mobility scale
C1	0	-	C	9.0	1.50	49.5	22.0	-	-

C2	0	-	C	10.0	1.53	52.0	22.2	-	-

P1	0	-	NS	15.3	1.23	36.3	24.0	115.8	1

P2	0	-	NS	13.1	1.25	29.5	19.0	93.6	3

P3	0	-	NS	10.9	1.15	34.0	25.6	50.7	5

P4	0	-	NS	13.0	1.20	28.4	19.7	99.0	8

P5	6	T9, R	PS	13.7	1.25	35.0	22.5	95.7	4

P6	10	T11, R	PS	11.8	1.28	43.3	26.4	35.7	1

P7	15	T11, L	S	14.8	1.41	38.0	19.3	79.6	6

P8	52	L3, L	S	11.8	1.48	45.0	20.7	5.5	8

When the MRI images were acquired, six of the DMD patients were still on DFZ but it had been ceased for P1 and P8, since 0.8 yr and 5.5 yrs respectively. See the Materials and methods section for information on the mobility scale.

The paraspinal muscles were imaged over the T8 to L4 region (9 cm above and 9 cm below T12-L1) where the apex of the scoliotic curvature in DMD patients is usually located. A 1.5 Tesla Symphony MRI Scanner (Siemens Medical Solutions, Malvern, PA, United States) was used with a 3D SE-T1 sequence (TR 550 ms, TE 14 ms, Nex 3) [15]. Slice thickness was 3 mm, the pixel dimension was 0.39 × 0.39 mm and the 12 bits image size was 512 × 512 pixels. A set of 63 images were collected with a 7 slices interleave to reduce the effect of the breathing and motion during MRI acquisition. The acquisition time, including subject preparation, ranged from 18 to 24 min. When a set of images had been acquired, each image within the set was rapidly inspected to detect if blurring occurred due to motion artefacts of the subject since no sedation was used. Following such verification, a new series of images had to be acquired for two patients.

The manual segmentation procedure used for back muscles was initially developed for the upper arm where segmentation in axial, then sagittal, and coronal planes was developed to minimize error in the detection of muscles boundaries. Validity of this procedure was assessed with two observers performing independently the segmentation of the same images and using two different image processing software. To assess the accuracy of the segmentation procedure, a MRI plexiglass phantom (cylinder of ∅ = 187 mm, 60 mm thick with holes diameters from 11.5 down to 1.1 mm) was used as well as MRI images obtained from the upper limb of normal subjects and from the erector spinae (ES) of adolescent scoliotic patients. The accuracy on a cross section area determination varied with the size of the object: for the phantom, it was 0.2% for its external diameter of 187.0 mm, 2.9% for a hole of 11.0 mm and 4.9% for 7.0 mm. A look-up table of the estimated volume versus the diameter of each hole was obtained. The segmentation accuracy was then estimated to be = 1% for back muscles (∅~30 mm) and = 0.9% for biceps brachii (∅~50 mm). Accuracy was also found quite dependent on image contrast than on the human factors involved in the segmentation [16]. In the present study with DMD patients, the data acquisition parameters offering the best contrast where chosen and the images were segmented by the observer who developed the technique (G.Z.). With a marching cube algorithm [17,18]. a faceted volumic model of the ES muscle and of the infiltrated fat was obtained. With the look-up table, the accuracy in the determination of the volume of the ES and its 3 constituents was estimated to be ≤ 1%.

Following a manual delimitation of the outline of the left and right erector spinae (ES) and IL, LO and SP muscles with SliceOmatic software (Tomovision Inc., Montreal), a threshold was experimentally set at 512 over 4096 gray levels within each closed contour for the automatic detection of the infiltrated fat on each slice. For each muscle, the cross section area of 6 to 8 consecutives slices, approximately the thickness of a vertebra, were used to get a volume associated to each vertebral level. As for the amount of fat infiltration in a muscle, it was expressed in percentage of that muscle's volume. For the controls and the NS patients, % values on the right were averaged as well as those on the left. Similarly, for the PS and S patients, an average for the convex side and one for the concave side was obtained. No distinction was made whether the spine deformation was on the right or on the left

The overlying skin thickness was measured on both sides of the spine from T9 to T11 over IL, LO and SP muscles. The cutaneous folds caused by creases in the sheet over the MRI platform that could be associated to muscular material, eliminated by averaging 9 measures made on each side of the folds where contrast between fat and muscle was low.

Due to a reduced number of patients, statistical analysis only included mean, standard deviation and linear correlation calculations.

## Results

**Figure 1:**
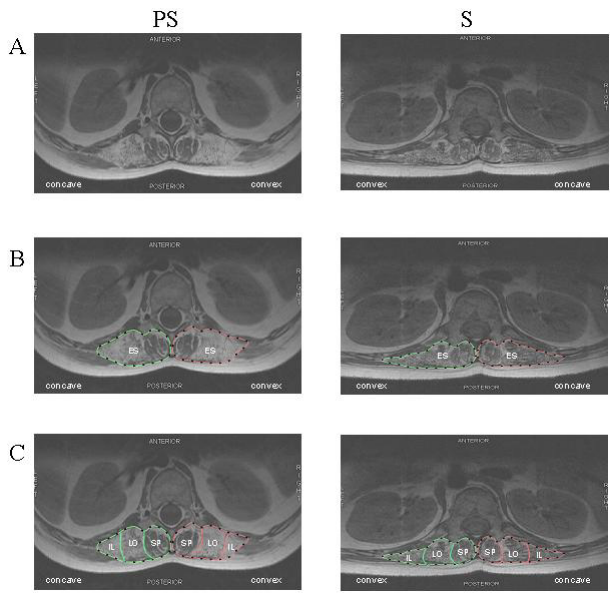
**MR images obtained at L2 level for the pre-scoliotic (PS) patient P6 (left column) and for the scoliotic (S) patient P7 (right column)**. A: original images; B: manually obtained contours of the erector spinae (ES); C: manually obtained contours of the spinalis (SP), longissimus (LO), and iliocostalis (IL).

A sample of the images of the trunk obtained from two patients is presented in Fig. 1. On those images obtained at L2, the contours of the IL, LO and SP muscles (making up the ES muscle) are outlined. Muscular volumes were obtained from successive images on which such contours had been outlined.

**Figure 2:**
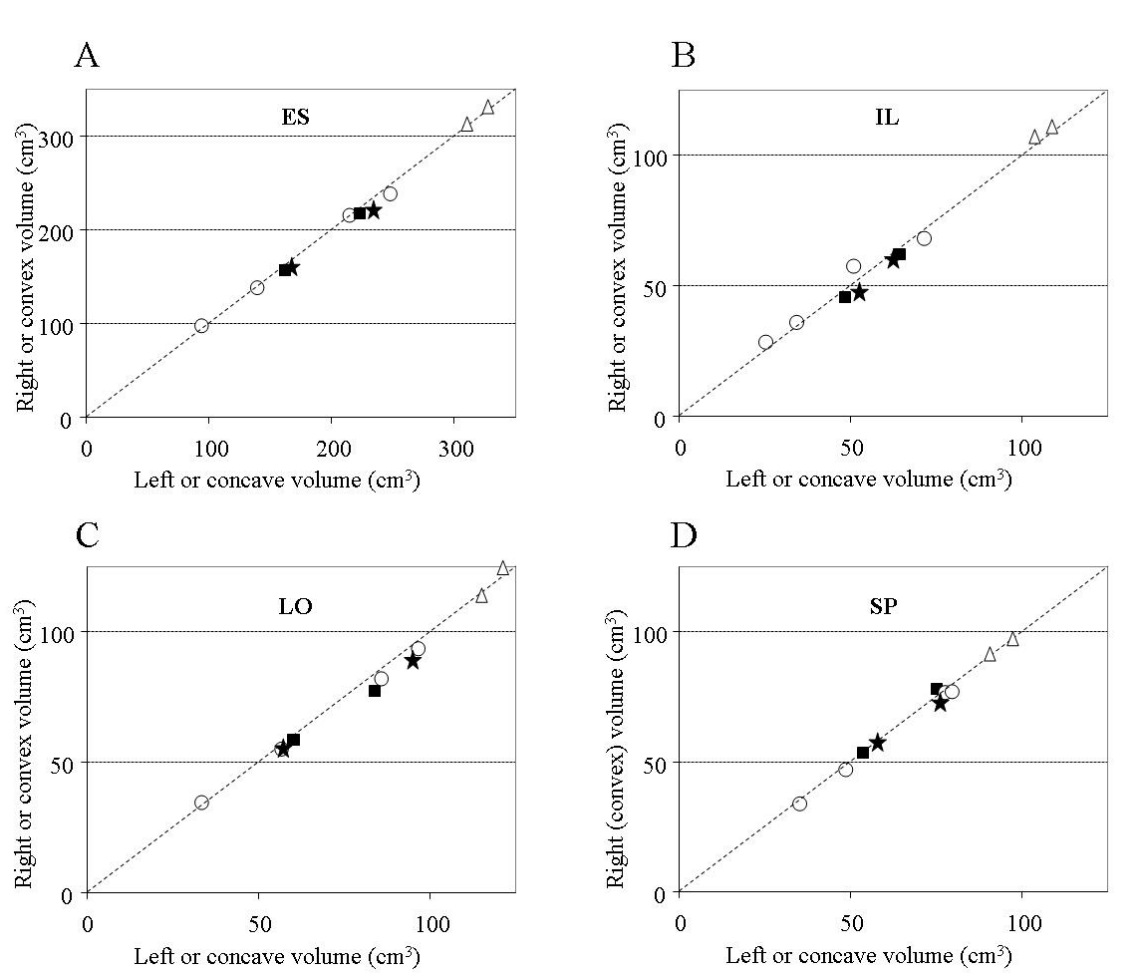
**Muscle volume measured on the left (concave side) versus on the right (convex side) of the spine between T8 and L4**. A: results for the erector spinae (ES). B: for the iliocostalis (IL); C: for the longissimus (LO); D: for the spinalis (SP). NS patients (o); PS patients (black square); S patients (black star); controls: (Δ). In each panel, the dotted diagonal indicates where equal values on both sides of the spine would fall.

Among the patients, the volume of ES muscle ranged from 95 to 243 cm^3 ^while it was over 300 cm^3 ^for the two controls (Fig. 2A). No correlation was found between the patients' ES muscle volume and their body mass index (BMI), neither with their age or height. For the 4 NS patients, the ES volume difference between right and left sides was 2.1 ± 1.5%. The difference was smaller for the 2 controls (1.4 ± 1.2%). For the PS and S patients, the volume of the ES muscle was smaller on the convex side by 2.8 ± 0.2% and 5.3 ± 0.7% respectively.

For the patients, the volume of the IL muscle ranged from 30 to 70 cm^3 ^and it was larger for the controls (Fig. 2B). For the NS patients, a larger volume of IL (7.5 ± 3.7%) was found either on the right or on the left. For the PS and S patients, the IL volume was larger on the concave side respectively by 4.8 ± 1.9% and by 7.8 ± 4.0%. The volume of the LO muscle was slightly larger than the IL (Fig. 2C) and for the NS patients, a larger IL volume of 3.4 ± 1.0% was generally found on the left. As for the PS and S patients, volume of this muscle was larger on the concave side by 5.3 ± 3.9% and 5.7 ± 2.1% respectively. As for the SP muscle (Fig. 2D), the difference in volume was 3.1 ± 0.9% for the NS. A larger volume of SP was found on the concave side of one PS patient and the two S patients but the difference (1.8 ± 1.3%) was similar between the two groups. Volume difference between patients and the controls was less important for the SP than for the IL and the LO muscles.

**Figure 3:**
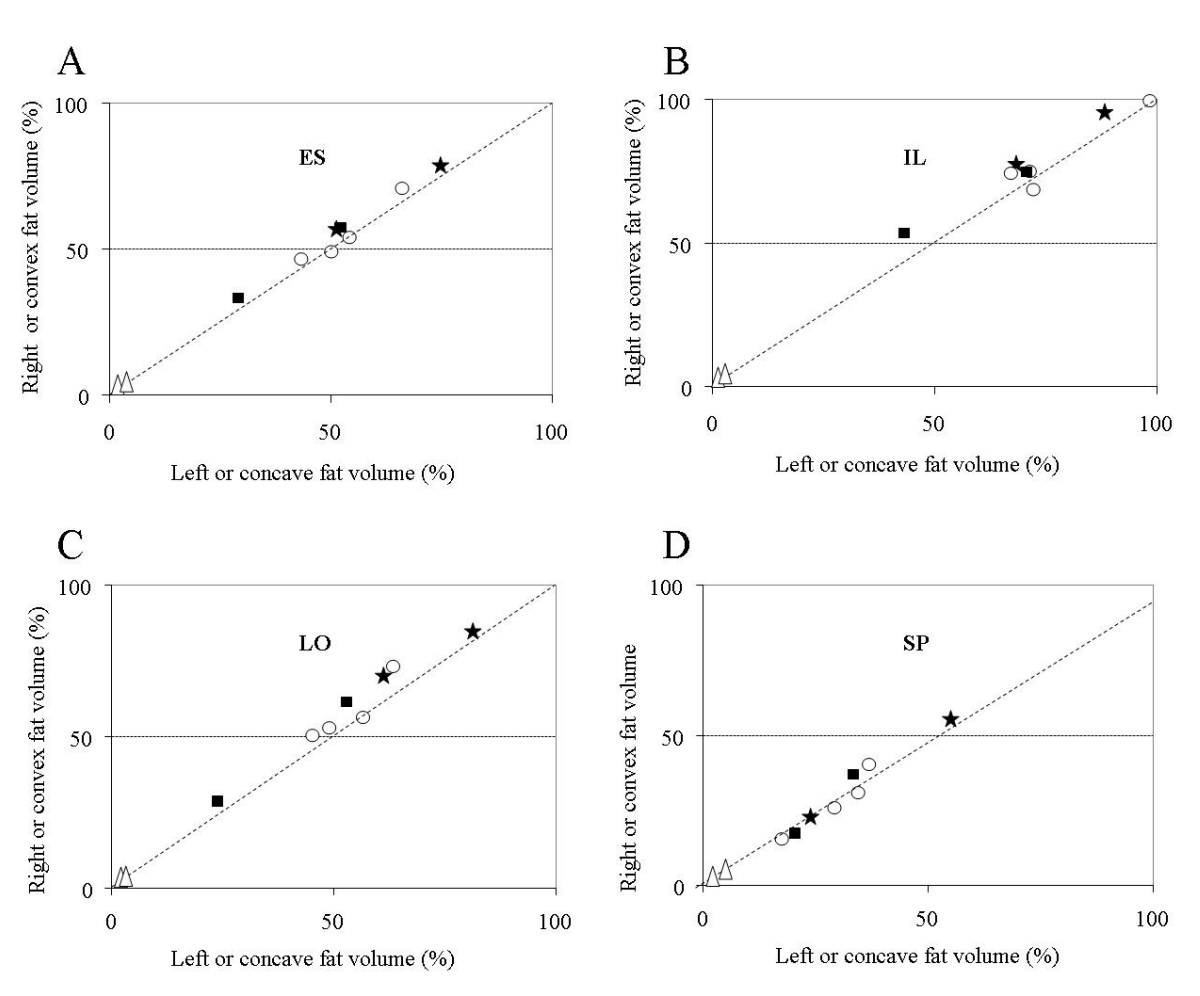
**Infiltrated fat on the left (concave side) versus on the right (convex side) of the spine between T8 and L4 for the ES muscle and for each of its constituent expressed in % of each muscle volume**. NS patients (o); PS patients (black square); S patients (black star); controls: (Δ). In each panel, the dotted diagonal indicates where equal values on both sides of the spine would fall.

Fat infiltration in the ES relative to its volume was much more important for the eight DMD patients (49.9% ± 1.6%) than for the two controls (2.6 ± 0.8%, symbol Δ in Fig. 3A). For the 8 patients, infiltration was more important laterally (i.e. IL, Fig. 3B) than medially (i.e. SP, Fig. 3D) and this translates in a mean fat/muscle volume ratio of 3.6 for the IL, 1.7 for the LO and 0.5 for the SP. Mean difference for the 2 controls was 1.4 ± 1.2%. For the ES (Fig. 3A), fat infiltration was larger on the convex side of both the S patients (symbols ⋆) and PS (symbols ■) by 4.4 ± 1.6% and 4.5 ± 0.7% respectively. In the IL muscle (Fig. 3B), the infiltration was larger for the S and the PS patients, on their convex side (7.9 ± 1.4 and 7.0 ± 4.8% respectively). For the NS patients, infiltration in the IL was larger either on the right or on the left (as in the ES). For the LO muscle (Fig. 3C), fat infiltration was larger on the convex side of the S (8.2 ± 0.9%) and for the PS patients (6.4 ± 2.5%); as for the NS, it was generally larger on the right side. A low level of infiltration was observed in the SP muscle (Fig. 3D) and, for 5 out of 8 patients, it was quite similar on both sides of the spine.

**Figure 4:**
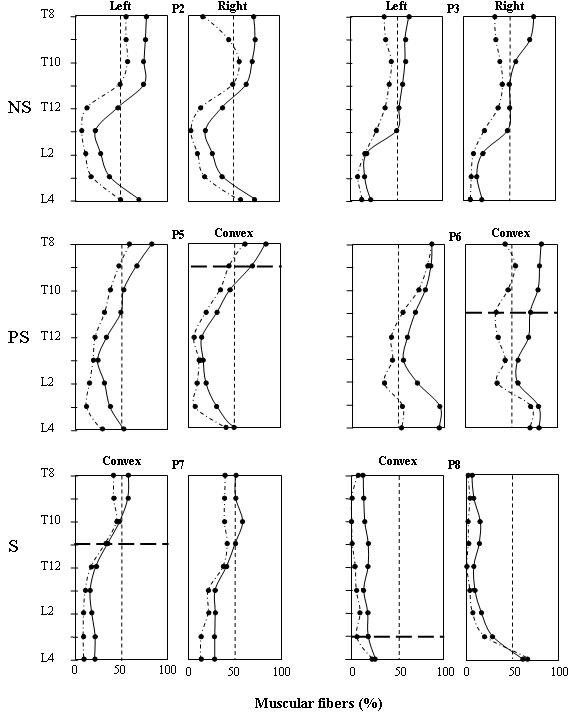
**Percentage of muscular fibers for the iliocostalis (IL: doted lines) and the longissimus (LO: continuous line) along the spine**. Upper panels: results on the left and right sides for non scoliotic (NS) patients P2 and P3. Middle panels: results on the concave and convex side for the pre-scoliotic (PS) patients P5 and P6. Lower panels: results on the convex and concave side for the scoliotic (S) patients P7 and P8. The thick horizontal dotted line on each convex panel indicates the apex position of the spinal deviations of PS and S patients.

**Figure 5:**
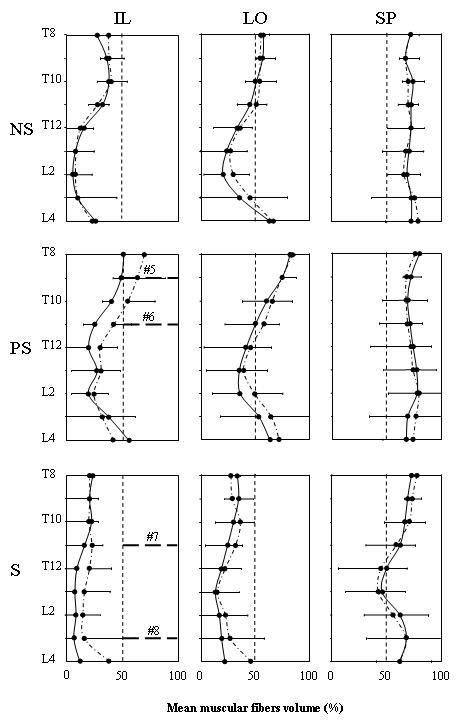
**Mean percentage (+ or - 1 standard deviation) of muscle fibers volume along the spine for the IL (left column), LO (middle column) and SP (right column) muscles**. Results for the NS, the PS and the S patients are respectively presented in the upper, middle and lower row. Within each group of patients, the continuous line represents the data on the right or convex side and the dotted line those on the left or concave side. On the middle and lower panels of IL, the thick horizontal dotted lines indicate the apex position of the spinal deviation of the PS (right convexity) and S patients (left convexity).

The muscle fibers volume obtained after removal of infiltrated fat in the IL, LO and SP was tracked along the imaged spine. Expressed in % (fibers volume/muscle volume ×100), the results obtained for the IL and LO are shown in Fig. 4 for six of the eight patients. It can be seen that the fibers volume of the IL and LO muscles tended to be minimal around L2. For PS and S patients, the fibers volume reduction of these 2 muscles was also important on the convex side along three vertebrae close to the apex of the deviation (horizontal dotted lines in Fig. 4). For the two PS patients, the mean infiltration level was 44.1 ± 16.3% vs 65.4 ± 15.0% for the two S patients. For P8 who had an important Cobb angle of 52°, loss of muscular fibers was important all along his spine especially on his convex side. For each group of patients, a mean fat infiltration value along the imaged spine obtained for the IL, LO and SP muscles are shown in Fig. 5. For the IL and LO muscles of the NS patients, the differences between the left and the right side were small while infiltration was larger on the convex side of the PS patients nearly all along the imaged portion of the spine. As for the spinalis muscle, its volume was similar in both PS and NS patients with a standard deviation larger for the PS patients. Concerning the S patients finally, fat infiltration in the IL and LO muscles was more important than for the PS and NS and it was larger on the convex side nearly all along the spine. As for the SP muscle, the level of infiltration is smaller than for the IL and LO and bears resemblance with the patterns of the spinalis muscles of NS and PS patients.

The overlying skin thickness was measured at T10 on each side of the spine. From the 9 measures taken on each side, the NS patients had a similar thickness on both sides of the spine (an average of 11.3 ± 4.0 mm for the right side and 11.2 ± 4.2 mm for the left side). As for the PS and S patients, thickness was larger on the concave than on the convex side (10.3 ± 6.3 vs 9.8 ± 5.6 mm and 14.8 ± 6.1 vs 13.5 ± 5.7 mm respectively).

## Discussion

Using MRI to study the back muscles of DMD patients, fat infiltration was found larger on the convex side of the deviations of pre- and scoliotic patients while no appreciable difference was observed in non-scoliotic patients. A different result was obtained by Stern and Clark [10] when they studied the back muscles of 16 scoliotic DMD patients with CT scans. They reported that fat infiltration was more important on the concave side of the spinal deviation but when their density readings (Table 2 of their paper) are closely scrutinized, one can be lead to consider that fat infiltration was larger on the concave side for only half of their patients. Also, their density readings were taken only at T9 and L3 levels in the medial (i.e. SP) and lateral (i.e. IL) portions of the ES and fat infiltration in the LO was probably not taken into account. In contrast, our data were continuously collected at every 3 mm from T8 to L4 and all the constituents of the ES muscle were analyzed. Through the reliability offered by the use of our 3D segmentation procedure, the accuracy of volume determination can be considered similar to the one of the cross section area (i.e. ≤ 1%). For the ES muscle of the two controls, the smallest differences in muscle volume was 1.4 ± 1.2% and for fat infiltration it was 2.6 ± 0.8% while for the DMD patients, these differences were larger. All of our results are then outside the segmentation accuracy. Segmentation was easier to achieve for ES due to its size and for SP because fat infiltration was minimal. For more severely infiltrated LO and IL muscles, an additional segmentation cycle (axial, sagittal, then coronal plane) was done compared to the number of iterations used to delineate ES and SP.

Some of the differences between our results and those of Stern and Clark may be associated to the effects of the DFZ: all of our patients had been and some were still treated (n = 6) with DFZ while none of their patients received such a treatment. DFZ has an overall positive impact on DMD patients' quality of life: it improves cardiac function, prolongs walking, and seems to eliminate the need for spinal surgery, although vertebral fractures and stunted growth occur [19]. Weight gain is a side effect of the medication but no correlation was found here between BMI and the duration of DFZ medication.

The presence of an uneven fat infiltration can induce a force unbalance on the spine which could lead to its buckling toward the weaker side. As the spine buckles, the skin becomes compressed on the concave side and stretched on the convex one. Our results indicate that the thickness difference was smaller for the PS than for the S patients (and absent for the NS patients). Due to the filtering effects of the overlying skin thickness, smaller EMG signals would be expected on the concave side. However, in another set of data collection with the same patients [20], a larger surface EMG signals were recorded on the concave side of the PS patients and on the convex side of a S patient. While smaller signals on the convex side of the PS may have result from the greater fat infiltration on that side, it cannot explain the larger signal on the convex of the S patient. It can thus be hypothesized that a more important neural input on one side of the spine can contribute to some extent to the presence of larger EMG signals on that side [21]. In our pre-scoliotic DMD patients, a deficit in muscle fibers on one side of the spine and an imbalance in the neural input could thus be two factors leading to the deformation of the spine.

As the Cobb angle gets larger than 15°, it seems that compensatory mechanisms to prevent further deviation of the spine are set in action. For instance, for scoliotic DMD P7, a larger EMG activity was detected on his convex side [20] as it is frequently reported in AIS [22,23]. The stretching of the skin on the convex side of a deviation causes a reduction in the overlying skin thickness, favoring the presence of a larger EMG signal but a greater fat infiltration on the convex side would counteract to some level such an increase. The presence of a larger EMG signal on the convex side may thus be attributed to a greater feedback from the more stretched muscle spindles and/or a larger neural input from the central nervous system. Imbalanced neural input was also considered to have a pathogenic importance in the etiology of AIS [21].

For Shimada [22], the trunk muscle imbalance would be one of the most important factors in the onset and progression of AIS since EMG amplitude is higher on the convex side when the deviation is progressive while no differences are observed in non progressive situation. According to the medical records of our PS and S patients, their spinal deformation did not progress over an 18 months period preceding our images acquisition. Either DFZ halted the progression of the deformation or their scoliosis was non progressive. Since higher EMG signals were still detected on the convex side, it seems that the scoliotic mechanism present in a DMD is somewhat different than in AIS.

To predict the progression of scoliosis in DMD patients, approaches such as Griffiths developmental scales, the Reynell language scales and the British picture vocabulary scales have been proposed [24,25]. but their reliability seems to be limited as compared to the vital capacity at age 10 when ambulation usually ceases [26]. As for Gibson and Wilkins [14], they proposed a clinical mobility index based on muscle weakness which is essentially caused by the progressive replacement of muscle tissue by fat. In our study, that mobility index was found correlated with fat infiltration in the ES (R^2 ^= 0.70, p < 0.01) but not with BMI nor with the age of the patients. This lack of correlation with age may be specific to the ES and its constituents since for thigh muscles (hip, mid-thigh, and knee), older boys demonstrated more prominent fatty infiltration than younger ones [27]. However, it has to be considered that despite a severe infiltration, NS P4, which had a very limited mobility, did not develop scoliosis since infiltration was similar on both sides of his spine. Therefore, an asymmetry in fat infiltration (Fig. 4) could help predict the development of a spinal deformation.

Only the ES muscle and its constituents were studied here since they can greatly influence the development of a scoliosis and their activity can be easily monitored from the skin surface. Investigation of less accessible trunk muscles, such as the abdominal and external oblique, psoas and quadratus lumborum, could be worth of investigation as they can also influence the stability of the spine.

DMD patients are frequently solicited to participate in a research protocol and we experienced difficulties with patients' recruitment. The small number of patients participating in our protocol constitutes the main limitation of the study. With fat infiltration appearing initially in the L2 region, images acquisition could be restricted in that zone thus reducing the time required for the execution of the MRI protocol. This could contribute to facilitate the recruitment of patients.

## Conclusion

In our pre- and scoliotic DMD patients, the volume of muscle fibers of the erector spinae was found smaller and fat infiltration was more important on the convex than on the concave side. Anatomically, the presence of less muscle fibers on one side of the spine due to a larger fat infiltration is a factor that can contribute to the application of uneven forces on the spine leading to its deformation. Physiologically, uneven forces could also result from the action of the central nervous system on those muscles and this action seems to be different between the pre-scoliotic and scoliotic stages. Since infiltration along the spine took place initially in the IL muscle around the L2 level and spread upward and downward towards the LO and the SP, any effort to halt such a progression could help improve the condition of the DMD patients. As for the overlying skin thickness, it was larger on the concave side of the deviation and this may help explain in part why a smaller EMG signal is generally recorded on that side. While the scoliotic mechanism present in a DMD share similarities with AIS, it also has differences. These preliminary results deserve further experimental work for confirmation.

## Competing interests

The authors declare that they have no competing interests.

## Authors' contributions

As a continuity of a previous study for the biometry of the upper limb muscles using MRI, PAM, CEA and GZ developed the present study to address the ES muscles' anatomy. PAM worked on the electromyography correlation, CEA addressed the biomechanical aspects. As for GZ, he developed the MRI sequence suitable for muscle biometry, the 3D segmentation procedure and segmented and analyzed all images. He also supervised the quality of the images during the MR acquisitions. All authors read and approved the final manuscript.

## Acknowledgements

The authors thank Dr. Decarie and the radiology department of Ste-Justine Hospital for their collaboration. Special thanks to Mrs. Diane Choquette and Ginette Labrecque for their help in the programming of the acquisition sequence. Many thanks to Mr. Christian Bellefleur, and Mrs. Julie Joncas, the research nurse responsible for patients' recruitment and to Mrs. Linda Pelletier for reviewing the text. Work supported by the Canadian Institutes of Health Research, the Natural Sciences and Engineering Research Council of Canada (156144-04) and le Fonds Québécois de la Recherche sur la Nature et les Technologies.
